# Global low-frequency motions in protein allostery: CAP as a model system

**DOI:** 10.1007/s12551-015-0163-9

**Published:** 2015-02-04

**Authors:** Philip D. Townsend, Thomas L. Rodgers, Ehmke Pohl, Mark R. Wilson, Tom C. B. McLeish, Martin J. Cann

**Affiliations:** 1 Biophysical Sciences Institute, Durham University, Durham, UK; 2 School of Biological and Biomedical Sciences, Durham University, Durham, UK; 3 Department of Chemistry, Durham University, Durham, UK; 4 Department of Physics, Durham University, Durham, UK; 5 Present Address: School of Chemical Engineering and Analytical Sciences, University of Manchester, Manchester, UK

**Keywords:** Protein, Allostery, Dynamics, Catabolite activator protein, Normal modes, Elastic network model

## Abstract

Allostery is a fundamental process by which ligand binding to a protein alters its activity at a distant site. There is considerable evidence that allosteric cooperativity can be communicated by the modulation of protein dynamics without conformational change. The Catabolite Activator Protein (CAP) of *Escherichia coli* is an important experimental exemplar for entropically driven allostery. Here we discuss recent experimentally supported theoretical analysis that highlights the role of global low-frequency dynamics in allostery in CAP and identify how allostery arises as a natural consequence of changes in global low-frequency protein fluctuations on ligand binding.

## Introduction

Allostery (from the Greek *allos stereos* “other solid”) is the regulation of protein function through the binding of an effector molecule at a site other than the protein’s functional site (Cornish-Bowden 2014). Allostery represents a fundamental mechanism that underpins normal and pathological molecular cellular processes (Motlagh et al. 2014). Despite its importance as a fundamental principle in biology, a generalizable and atomistic description of allostery is lacking. Early models of allosteric co-operativity were rationalised through the description of distinct protein conformers. In the ‘symmetry model’ (MWC: Monod-Wyman-Changeux model), an equilibrium between two conformational states is perturbed by ligand binding such that all subunits in the oligomeric protein change state in a concerted fashion (Monod et al. 1965). In the ‘sequential model’ (KNF: Koshland-Nemethy-Filmer model), a requirement for conformational symmetry is replaced with a strict application of ‘induced-fit’ binding of ligand with distinct protein conformers in the bound and unbound states (Koshland et al. 1966). These influential models facilitated the development of a view of allostery dominated by a view that structural change was the underlying mechanism. More recently, however, the direct role of structural fluctuations in allostery has become increasingly apparent.

## Protein dynamics in allostery

A theoretical study of Cooper and Dryden in 1984 was an effective challenge to predominant view of allosteric control through structural change (Cooper and Dryden 1984). Their statistical thermodynamic analysis demonstrated that allosteric interaction free energies of the order of several kJ mol^−1^ could be achieved through ligand induced changes in protein dynamics. Specifically, the dynamic allostery described is achieved primarily through altered entropy and was hypothesised to arise from the transformed frequency and amplitude of protein thermal motion. At its broadest level, the idea that proteins have evolved to take functional advantage of not only the mean conformation but also the inherent thermal fluctuations about this mean was introduced. It was found that root mean square fluctuations around the same average position of only 1 % per atom, when summed over an entire protein, could permit changes to the allosteric free energies purely through alterations in entropy (Cooper and Dryden 1984). Numerous recent observations have supported the idea that protein dynamics is linked to evolutionary selection pressures. For example, analysis of a large dataset of protein structures has demonstrated that protein backbone flexibility is well conserved at family and superfamily levels despite the lack of obvious primary sequence similarity (Maguid et al. 2006). This conservation was subsequently proposed to arise from a conservation of the lowest frequency global protein motions as identified through a direct comparison of protein pairs (Maguid et al. 2008). These studies link well with the hypothesis that the lowest frequency modes are more evolutionarily robust through their link to protein functional motions (Nicolay and Sanejouand 2006; Tama and Sanejouand 2001). For example, evolutionarily conserved low-frequency deformation lobes have been observed to underpin distinct functions within the small monomeric G-proteins (Raimondi et al. 2010). Subsequent theoretical developments have more specifically emphasised the role of ligand-modulated low-frequency global modes of protein dynamics in generating allosteric interaction between distant binding sites (Hawkins and McLeish 2004, 2006a). Following from this theoretical analysis, recent developments in nuclear magnetic resonance (NMR) spectroscopy has revolutionised our experimental capacity to study the role of protein structure, dynamics and thermodynamics in allosteric systems (Manley et al. 2013). A particularly valuable development has been the use of relaxation NMR methodologies that use fast internal protein dynamics as a proxy for changes in conformation entropy on ligand binding (Wand 2013). It is become clear, therefore, that dynamics has a crucial role to play in allostery.

## The role of dynamics in allostery in the catabolite activator protein

**Fig. 1:**
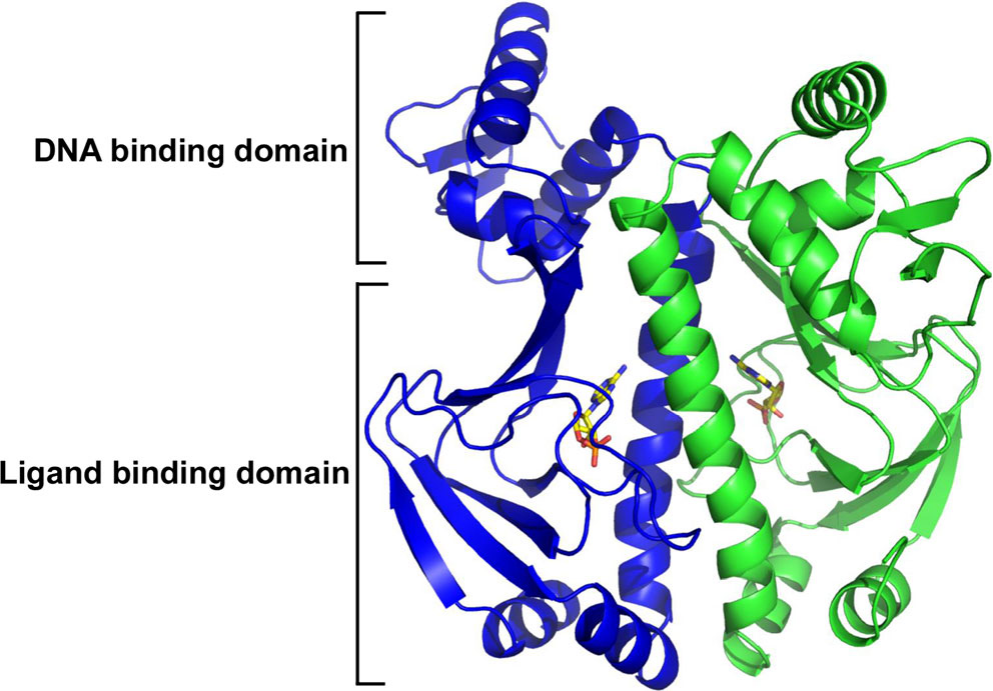
Ribbon diagram of the crystal structure of CAP determined at 1.48 Å resolution (PDB 4HZF) showing the secondary and tertiary structures of the CAP homodimer with cAMP bound

While it was evident that dynamic effects were likely to play an important role in some cases of allostery, there was no direct experimental evidence to suggest that dynamics could be the dominant underpinning mechanism in an allosteric system. Work from the laboratory of Kalodimos in 2006 took advantage of the strongly negatively allosteric regulation of a truncated variant of the Catabolite Activator Protein (CAP) to examine the possibility of dynamics-driven allostery (Popovych et al. 2006). CAP is a 210 amino acid homodimeric transcription factor of *Escherichia coli* that binds cAMP generated by adenylyl cyclase in response to the phosphorylated form of Enzyme IIA^Glc^ (phosphorylated in response to the phosphoenolpyruvate–carbohydrate phosphotransferase system) (Gorke and Stulke 2008). CAP consists of two domains per monomer. The N-terminal 138 amino acids represent the ligand-binding domain while the C-terminal portion of the molecule represents the DNA binding domain (Fig. 1). A range of elegant NMR studies has shown that conformational entropy via altered backbone and side-chain dynamics is linked to positive allostery between ligand and DNA binding where cAMP binding promotes the interaction with DNA (Tzeng and Kalodimos 2009, 2012). Negative allostery also exists in native CAP between the cAMP binding sites and is of the order of *K*_2_/*K*_1_ = 1.7 (where *K*_1_ and *K*_2_ represent the dissociation constants for the first and second cAMP binding events); however, *K*_2_/*K*_1_ ≈ 100 for a CAP variant representing only the first 138 amino acids (the ligand binding domain). This enabled the apo-, single ligand bound, and double ligand bound CAP states to be isolated and analysed by NMR and isothermal calorimetry (ITC) to assess the role of dynamics in allosteric regulation. It was demonstrated that there were minimal changes in protein structure between the single and double liganded proteins. Rather there were distinct changes in protein motions between states and the enhanced negative co-operativity was driven by changes in entropy. Binding of the first molecule of cAMP had little effect on motion in the ps–ns range but activated slow motions (μs–ms range) across both subunits (Popovych et al. 2006). Binding of the second molecule of cAMP suppressed both fast and slow motions. The suppression of the μs–ms range motions on binding the second molecule of cAMP represents the entropic penalty that is the source of negative co-operativity and the study overall demonstrated that allostery can arise through a purely dynamic mechanism.

## Normal mode analysis

A significant issue arises with understanding the theoretical underpinning of allostery in CAP as a number of hypotheses have been presented to explain the mechanism by which dynamic fluctuations are communicated between allosterically coupled sites in various protein systems. One hypothesis is that ligand binding in an allosterically regulated system alters the structure of the global normal modes and therefore the coupling interaction between allosteric sites. This changes the ensemble of the protein structures, incorporating the allosteric sites, and therefore the free energy of binding (Hilser et al. 2012; Liu et al. 2007; Motlagh and Hilser 2012). An alternative proposal has discussed physically connected pathways of activated or repressed dynamics between allosteric sites (Reynolds et al. 2011; Zhuravleva et al. 2012; Zhuravleva and Gierasch 2011). A third view has proposed that large-scale motion dispersed across the protein, the normal modes, can carry the allosteric signal without a requirement for structural change (Hawkins and McLeish 2004, 2006b, c). The normal modes describe the different harmonic vibrational oscillations (typically over damped in the case of the slowest modes) in a protein around a mean minimum energy structure. Normal mode analysis (NMA) represents a major simulation technique to study large-scale motions in proteins (Brooks and Karplus 1983; Go et al. 1983). NMA typically requires a set of Cartesian co-ordinates from an X-ray determination of protein structure and a force field that describes the interactions between the atoms. The calculation involves first, the minimisation of the conformational potential energy, second, calculation of the “Hessian” matrix from the second derivatives of the potential energy with respect to the mass-weighted atomic co-ordinates and, finally, diagonalisation of the Hessian matrix to yield the eigenvalues and eigenvectors that compose the normal modes. In proteins, the low-frequency modes are typically associated with function through collected motion while the more localized high-frequency modes are spatially localised due to dynamic disorder and are associated with protein disorder (Bahar et al. 1998). NMA of an all-atom protein structure represents a significant computational problem, particularly due to the long time scales required for a full analysis of the motions associated with the low-frequency modes. One approach to circumvent this problem is the use of elastic network models (ENM) in which the total number of atoms in the protein is reduced to only Cα atoms connected by a network of elastic Hookean springs (Tirion 1996). ENM-based models reproduce low-frequency modes that compare well with experimentally derived data (Delarue and Sanejouand 2002; Valadie et al. 2003). Numerous software packages now permit the calculation of ENMs including elNémo, PDBMAT, ProDy and MAVEN (Bakan et al. 2011; Suhre and Sanejouand 2004; Tirion 1996; Zimmermann et al. 2011). Coarse-grained models can therefore provide a reasonably accurate calculation of dynamics over the long time scales required. A very coarse-grained normal mode analysis of internal monomer modes coupled across a dimer interface was able to confirm qualitatively that a fluctuation–allostery model was capable of capturing the negative cooperativity between the two cAMP binding sites of CAP. An increase in internal entropy on the first and a decrease on the second binding event is possible even when identical local changes occur (Toncrova and McLeish 2010).

## The normal modes in dynamic allostery in CAP

**Fig. 2:**
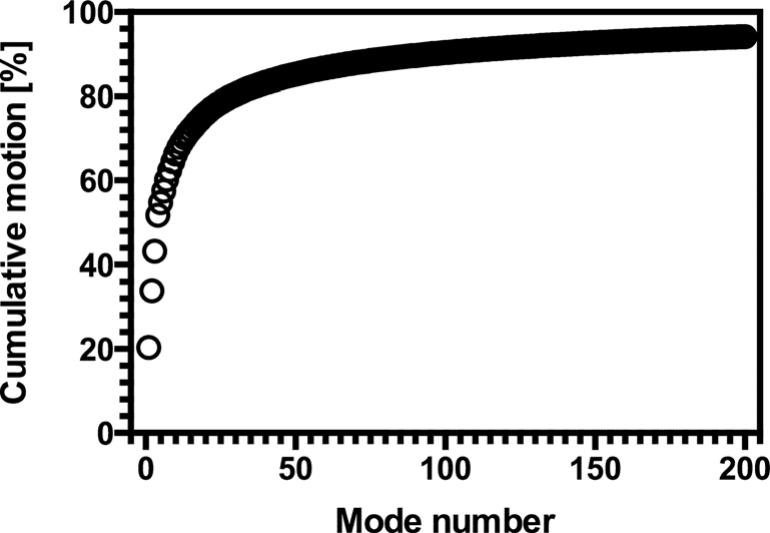
Mode number for an ENM for CAP plotted against percentage total cumulative protein motion for the first 200 modes

**Fig. 3:**
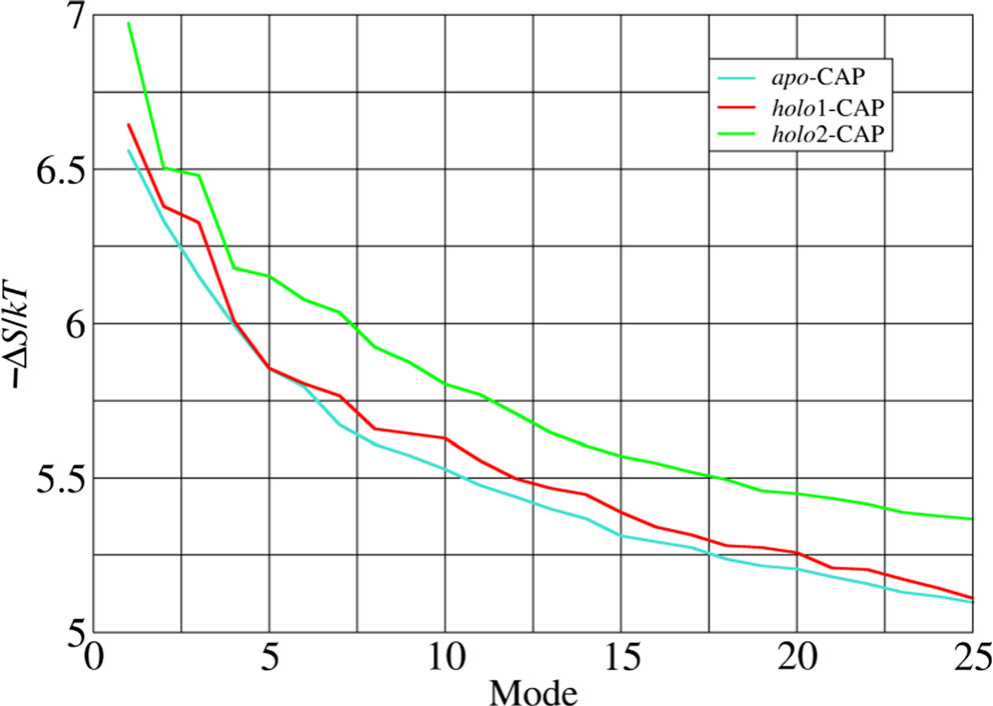
Mode number for an ENM for CAP plotted against entropy (Δ*S*/*kT*) for the first 25 modes. Apo-CAP (*blue*), CAP bound with one cAMP (holo1-CaP) (*red*), and CAP bound to two cAMP molecules (holo2-CAP) (*green*)

**Fig. 4:**
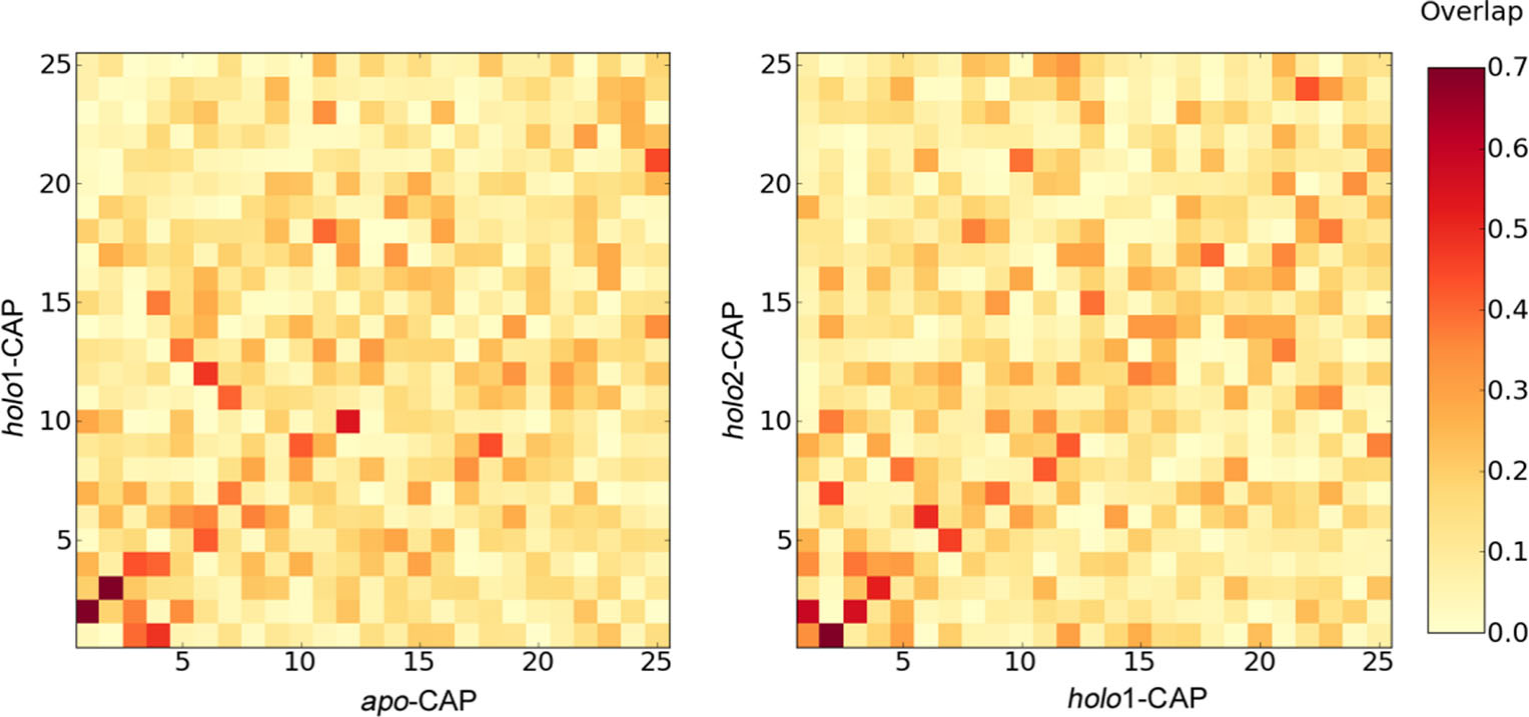
The similarity in eigenvectors between *apo*-CAP and *holo*1-CAP (*left*) and *holo*1-CAP and *holo*2-CAP (*right*). The first six rotational and translational modes are not represented. The *colour chart* represents the degree of overlap in eigenvector

Rodgers et al. (2013a, b) explored in more detail the suggestion that modulation of the normal modes of protein motion was the underpinning molecular mechanism that described dynamic allostery in CAP. The authors therefore applied coarse-grained modelling through ENMs to investigate the entropic contribution of cAMP binding to allostery in CAP. The allosteric free energy for binding the second molecule of cAMP in the calculated ENM was remarkably similar to experimentally obtained values, indicating that the normal modes could successfully recapitulate allostery in CAP. The value of *K*_2_/*K*_1_ (where *K*_2_/*K*_1_ represents the ratio of the second to first dissociation constants for cAMP) obtained for independent wild-type ENMs (*K*_2_/*K*_1_ = 1.35) was similar to that obtained experimentally through isothermal titration calorimetry (*K*_2_/*K*_1_ = 1.68 ± 0.04 SEM, *n* = 32). The value of *K*_2_/*K*_1_ was independent of spring strength and mode frequency. For example, varying uniform spring strength throughout the ENM eight-fold gave only an approximately 1.5 % change in *K*_2_/*K*_1_ (Table 1). A spring distance cut-off (8 Å) was selected that gave an optimal correlation value for B factors between the calculated ENM and the CAP crystal structure, despite the fact that a poorer correlation associated with higher values for *K*_2_/*K*_1_ (Table 2). Cut-off values at 7.5 Å and less gave calculated models with the atoms improperly connected. The ENM therefore captures negative allostery in CAP. The value of *K*_2_/*K*_1_ reported for the ENM was derived from where values of *K*_2_/*K*_1_ converged with increasing mode number (typically over 75 modes). Analysis of the cumulative protein motion in the CAP ENM reveals that the first 75 modes represent approximately 90 % of the total motion within the model (Fig. 2). The allostery captured by the ENM is therefore represented by the lower-frequency modes that represent the majority of the motion in CAP. Further, an analysis of just the first 25 modes, representing 78 % of the total protein motion, within 16 ENMs derived from independent X-ray CAP structures is still able of capturing a significant degree of negative allostery (*K*_2_/*K*_1_ = 1.13 ± 0.01). Figure 3 shows the entropy of the first 25 modes in such a CAP ENM, and reveals the source of the negative allostery. The binding of the first molecule of cAMP (*holo*1-CAP) to unliganded CAP (*apo*-CAP) results in an increase in entropy. Binding of the second molecule of cAMP (*holo*2-CAP) shows an enhanced unfavourable entropy change and represents the source of negative allostery. It is of note that the relative entropy increase on binding the second molecule of cAMP is a cumulative effect over the whole 25 modes examined and is not due to a change in one or two dominating modes. An examination of the similarities between eigenvectors further supports the finding that cAMP binding causes generic changes to motion in CAP (Fig. 4). A comparison of both *apo*-CAP/*holo*1-CAP and *holo*1-CAP/*holo*2-CAP shows that the lowest frequency modes, which typically represent motions of the DNA binding domain, are unaltered albeit slightly re-ordered. In contrast, the higher modes are directly altered in form, demonstrating that cAMP binding generically alters motion within CAP and supporting the observations of entropy increases per mode of Fig. 3.

**Table 1:** Variation in allosteric co-operativity (*K*_2_/*K*_1_) with ENM spring strength (*k*)

*k*	*K*_2_/*K*_1_	% change in *K*_2_/*K*_1_ from *k* = 1
0.5	1.356	0.460
1.0	1.350	0
2.0	1.370	1.486
3.0	1.360	0.725
4.0	1.361	0.839

**Table 2:** Variation in allosteric co-operativity (*K*_2_/*K*_1_) with ENM spring distance cut-off (Å)

Cut-off (Å)	*K*_2_/*K*_1_	*R* ^2^ between B factors for ENM and crystal structure
7.0	0.468	Not fixed
7.5	0.320	Not fixed
8.0	1.350	0.804
8.5	1.391	0.622
9.0	1.429	0.618
9.5	1.880	0.609
10.0	2.549	0.589

**Fig. 5:**
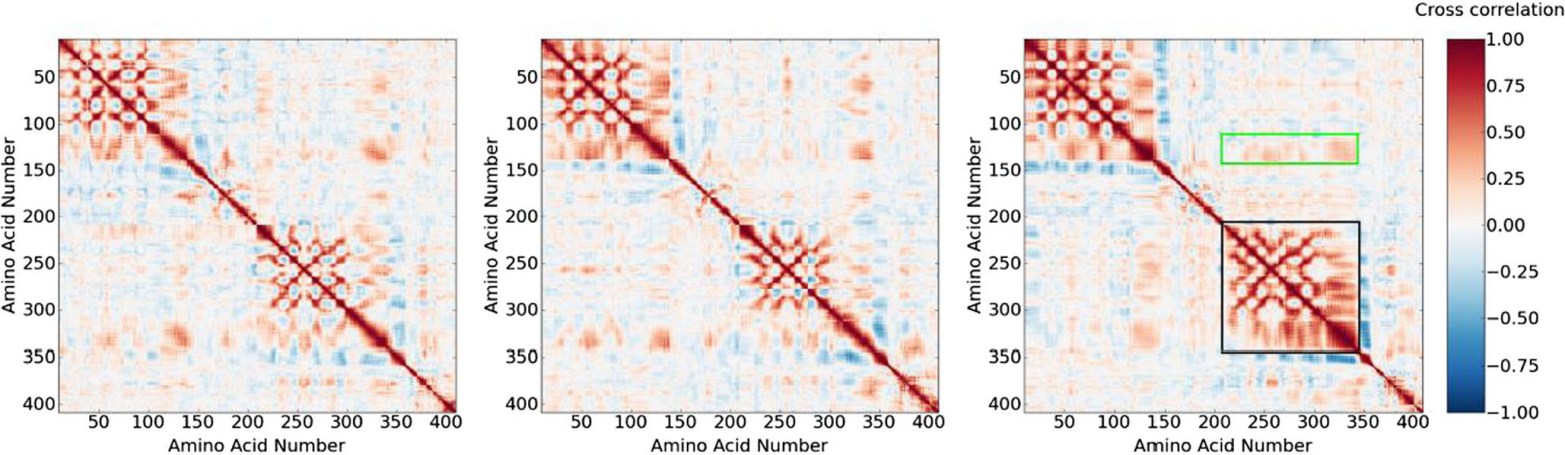
Correlated motions in ENMs for *apo*-CAP (*left*), *holo*1-CAP (*centre*) and *holo*2-CAP (*right*). Both the *x* and *y* axes represent amino acid number in CAP for the first (1–210) and the second (211–420) cAMP binding monomer. The *colour chart* represents the degree of correlation and anti-correlation in motion

The ENM therefore provides significant insight into the mechanism through which allostery in CAP arises through alterations to the normal modes. Allostery in the CAP homodimer is explained, through contributions to binding entropy, if there are correlations in the low frequency motions between CAP monomers and ligand binding modifies this correlation (Balabin et al. 2009). The modulation of these correlated motions within the ENM can be further investigated. Figure 5 shows an analysis of correlated motions within ENMs for *apo*-CAP, *holo*1-CAP, and *holo*2-CAP. Binding of both the first and second molecules of cAMP influences correlated motions throughout CAP. A particular significant change of relevance to cooperativity in CAP is that binding of the second molecule of cAMP not only enhances the degree of correlated motion within the ligand binding domain of the second CAP monomer (see black box in Fig. 5) but also enhances the degree of correlated motion between opposing ligand binding domains, particularly at the dimer interface (see green box in Fig. 5).

## A mutational map for dynamic control of allostery in CAP

**Fig. 6:**
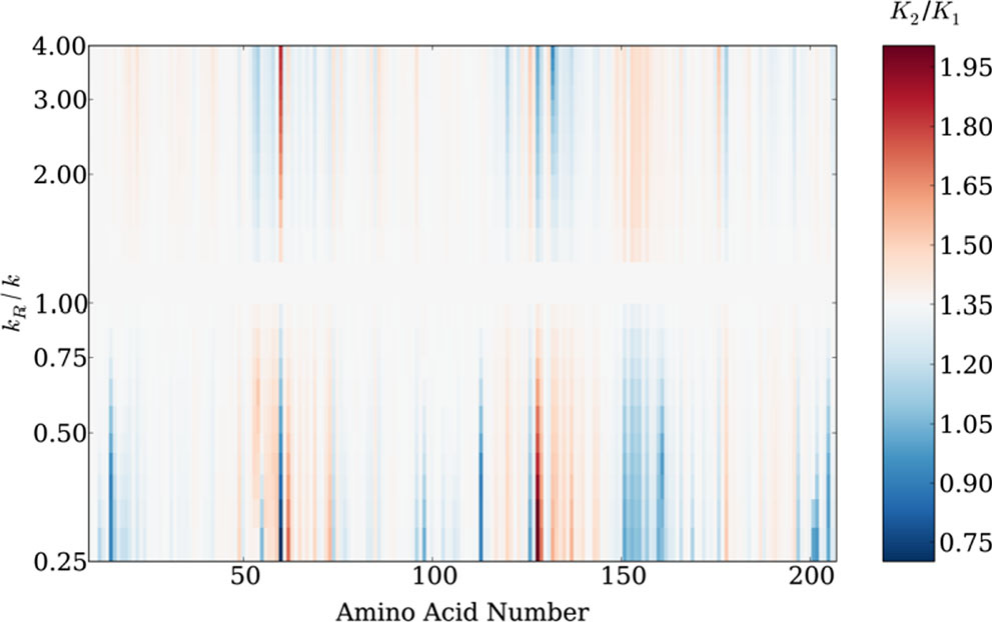
Global map for an ENM for CAP plotting amino acid number for the CAP monomer and dimensionless change in *k*_R_/*k*. The *colour key* represents the change in the ratio of the second to first dissociation constants for cAMP. *White* corresponds to values of *K*_2_/*K*_1_ predicted by the wild-type ENM. *Red* corresponds to increased values of *K*_2_/*K*_1_ (increased negative co-operativity) and *blue* to decreased values of *K*_2_/*K*_1_ (decreased negative co-operativity and positive co-operativity). The figure represents the influence of alterations in the stiffness of the spring constant (*k*_R_/*k*) at every amino acid throughout CAP with the resulting influence on *K*_2_/*K*_1_. The *y* axis represents 22 equal step changes in values of *k*_R_/*k*. Figure taken from Rodgers et al. (2013b)

Changes to the force constants that control the connectivity within the CAP ENM could be reasonably expected to influence the changes in correlated motion and total entropy, and thus allostery, on cAMP binding. At the level of the ENM altered amino acid side chain interactions are modelled by altered spring strengths between the corresponding Cα and its neighbours. As the ENM is a computationally efficient method for calculating ΔΔ*G*, it was possible to vary the elastic force constant at every Cα atom in turn and calculate the effect on allosteric connectivity. At each site, the spring constant (*k*_R_/*k*; corresponds to *k*_amino acid number_/relative spring strength and *k*_R_/*k* = 1 throughout the wild-type ENM where the initial model used uniform spring constants) could be increased (to represent a strengthened side chain interaction) or decreased (to represent a weakened side chain interaction) and ΔΔ*G* calculated. The resulting allosteric map, a two-dimensional colour-coded surface that related *k*_R_/*k* for every amino acid to *K*_2_/*K*_1_, could reveal features of the protein that exerted control over allostery (Fig. 6). A cursory examination of Fig. 6 reveals that residues that exert an influence over allostery in CAP are distributed over the entire CAP monomer but also with a tendency to form clusters (e.g. at the dimer interface). This gives the appearance of “control at a distance” whereby modifications in the ENM modulate allostery through changes in binding entropy through a global change in the normal modes. It is possible to think of these controlling residues operating as a “second order” allosteric interaction; they control, at a distance, the strength of an existing allosteric interaction between two other sites.

A key feature of the ENM for CAP and the insight obtained into the role of low frequency global motions in allostery is that it enables clear predictions for experimental validation. The global allosteric control map for the ENM (Fig. 6) enabled the selection of allosteric control residues for further testing through the introduction of mutations into the CAP protein and an assessment of allostery by isothermal calorimetry (ITC). Isothermal calorimetry represents a standard experimental tool for the experimental assessment of the thermodynamics of protein ligand interactions that directly calculates *K*_a_, Δ*H*, and binding stoichiometry and subsequently Δ*G* and Δ*S* from the Gibb’s equation (Brown 2009). Rodgers et al. 2013b selected amino acids for experimental analysis using a range of criteria:

1. Amino acids were selected that could enable both strengthening and weakening of interactions at a single site through altered hydrophobic interactions or at sites where electrostatic interactions could be altered without any adverse influence on local side-chain bulk.
2. Sites were selected at both key control hotspots (e.g. at the central helix) and away from any presumed direct spatial trajectory between cAMP binding sites.
3. Sufficient sites were selected such that observed correlations between experimental and theoretical data were unlikely to have arisen by chance.

Sites at the CAP dimer interface (V132) and on a surface loop distant from both the cAMP binding site and the dimer interface (H160) that were predicted to modulate allostery on side-chain mutation. An additional site (V140) was selected as it was predicted to have no influence on allosteric control. It was observed that mutations predicted through the ENM to increase the extent of negative co-operativity (V132A), decrease the extent of negative co-operativity (H160L), or switch allostery in CAP from negative to positive co-operativity (V132L), all behaved as expected when analysed experimentally. In each case, there was a semi-quantitative relationship between the degree of change in *K*_2_/*K*_1_ predicted by the ENM (Fig. 6) and that observed experimentally. X-ray structure analysis of all protein variants demonstrated that this rational engineering of altered allostery occurred in the absence of any significant change in overall protein structure. At the predicted V140 neutral site, a V140L mutant behaved as predicted by the ENM with no significant change in *K*_2_/*K*_1_. The V140A mutation caused a side-chain rotation of C179 to generate a new contact as shown by crystal structure analysis. Experimental findings for V140A were as predicted when this contact was strengthened in the ENM calculations. To summarize, therefore, all mutations tested qualitatively matched the predictions made by the ENM and included even a controlled change in the sign of allostery from a negatively to a positively allosteric protein. These findings were further extended by an analysis of the CRP/FNR transcription factor family member GlxR of *Corynebacterium glutamicum*. The role of the normal modes in the control of allostery in CAP was also observed to hold true in GlxR and was supported experimentally (Rodgers et al. 2013b; Townsend et al. 2014).

A subset of amino acids in CAP is therefore predicted to exert control over allostery and the data for several of these are experimentally supported. This allowed a test of the hypothesis that amino acids that exert control over allostery would be more invariant in related proteins from different species, presuming that allostery confers a selective advantage on the organism. Such an analysis is complicated as different but overlapping subsets of amino acids might confer a selective advantage for their contribution to ligand binding and the mean structure, in addition to allosteric control. Notwithstanding this caveat, an examination of 163 CAP variants from diverse bacterial species demonstrated that the rate at which an amino acid mutates was negatively correlated with *K*_2_/*K*_1_. Residues that contribute to low-frequency fluctuations in CAP-like proteins are therefore linked to evolutionary selection pressures.

## Global protein properties for dynamic control of allostery

**Fig. 7:**
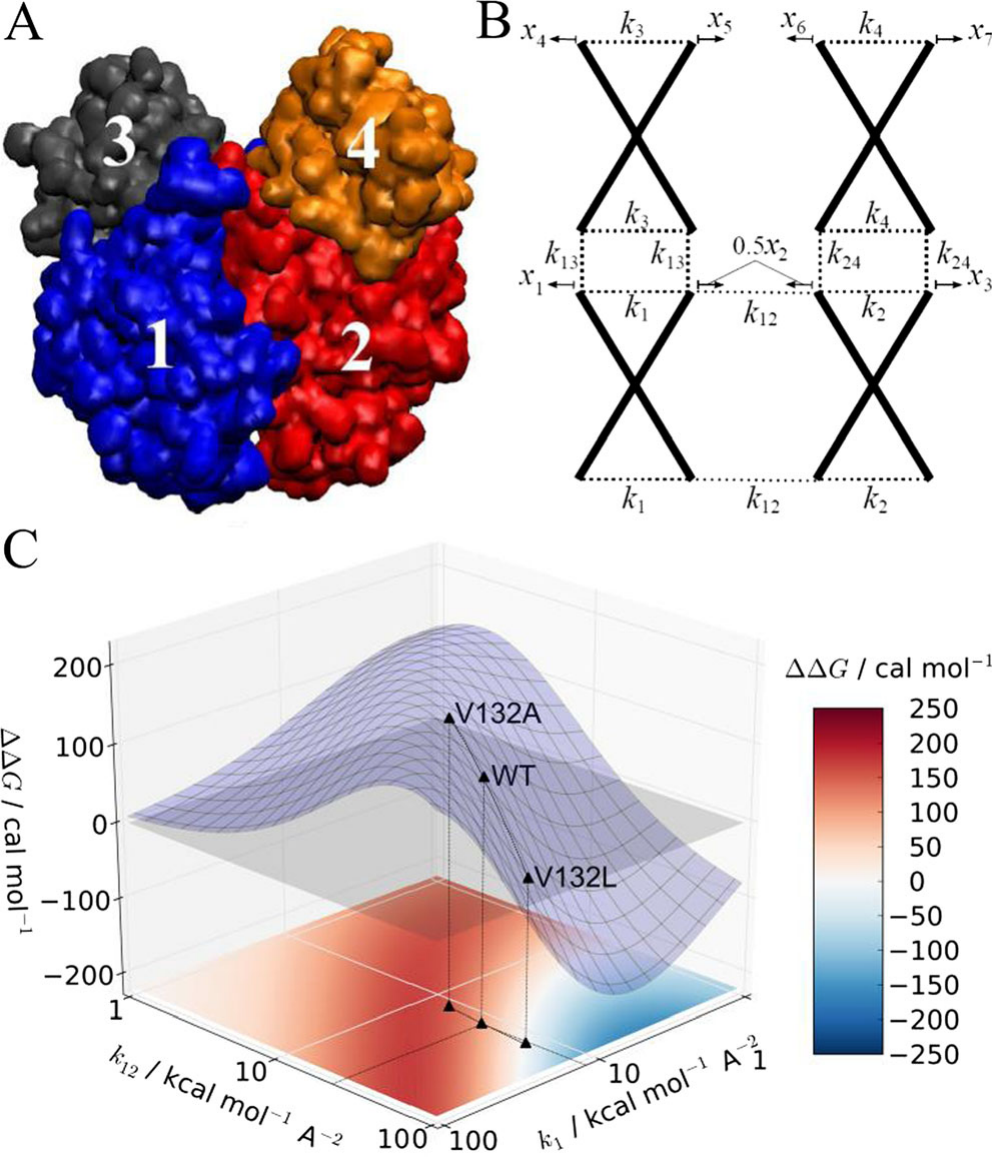
A super-coarse-grained models for CAP. **a** The elastic block representation for CAP that emerges from constraining all residues whose relative spatial fluctuations are less than 3 Å into a single rigid domain. **b** The corresponding model in which each block is accorded a single internal mode. For the super-coarse-grained CAP dimer, the internal subunit coupling strengths are characterized by *k*_1_ though *k*_4_ and the intersubunit couplings by *k*_12_, *k*_13_, and *k*_24._ Coupling strengths were defined by principal component analysis of 300-ns simulations for the no ligand, single ligand, and double ligand bound states. **c** Allosteric free energy in CAP as a function of the reduced two-dimensional landscape derived from the effective internal elastic strength within the cAMP binding domain (*k*_1_) and the inter-dimer elastic strength (*k*_12_) of the super-coarse-grained model. Figure taken from Rodgers et al. (2013b)

The theoretical analysis of allostery in CAP discussed here is suggestive of general underlying biophysical principles that permit allostery. The route taken to understand these principles has been one of *simplifying* the CAP protein for analysis (i.e. through the use of the ENM) rather than attempting to isolate such principles from fully atomistic representations of the protein. This approach can be carried further by coarse-graining CAP into a rotational-translational block representation (Durand et al. 1994). In such a representation, CAP is modelled as two coarse-grained blocks per monomer (one for the cAMP-binding domain and one for the DNA-binding domain) (Fig. 7a). Each individual block is rigid in this model, with internal residues fixed in relation to one another but with an integrated coupling strength. The blocks are connected to their neighbours by the remaining springs of the ENM (Fig. 7b). The rigid blocks are permitted one internal breathing motion and are permitted to move relative to each other. The justification for this approximation is the observed large degree of elastic inhomogeneity within and between the blocks. Remarkably, negative allostery can even be captured in such a super-coarse-grained model system for CAP (*K*_2_/*K*_1_ = 1.08) (McLeish et al. 2013). The actual position of wild-type CAP, or its variants, represent single points on a larger allosteric free energy landscape, and the position of any CAP variant is predominantly determined at this level of coarse-graining by changes to the coupling strength within the ligand-binding domain (*k*_1_) and between monomers (*k*_12_). (Fig. 7c).

The description of negative allostery in a super-coarse grained model for CAP raises an even more fundamental question. What are the required properties of a protein that can permit low-frequency dynamics controlled allostery to arise? McLeish and co-authors performed a series of calculations to exemplify the fundamental properties of fluctuation-induced allostery (McLeish et al. 2013). While this approach does not serve to illustrate any particular protein per se, it does function to identify key biophysical requirements that must be fulfilled to permit allostery to occur. The highly coarse-grained “toy” systems analysed revealed that a key requirement for fluctuation-induced allostery to occur is an inhomogenous elastic density in the system. In a protein, such elastic inhomogeneity can arise through both relatively rigid and less rigid regions within the internal environment of the protein and also through the boundary geometry of the folded protein (as observed at a simple level in the super-coarse-grained model for CAP). Significantly, dynamically induced allostery that operates through modulation of the normal modes has the potential for being either positive or negative in sign so long as there is at least two coupled degrees of freedom within the system. This theory therefore suggests that the requirements for an appropriately tuned internal elastic architecture that supports the functional requirements for allostery in a protein can co-exist with the structural requirements that underpin other aspects of protein function.

## Conclusions

The CAP protein of *E. coli* has served as an excellent exemplar to demonstrate how thermal excitation of biomolecular normal modes can underpin allosteric cooperativity. Analysis of the theoretical framework for allostery in this model has allowed both the rational engineering of allostery and the identification of fundamental protein properties that permit control of allostery through the normal modes. CAP represents a case of dynamic allostery that can be altered through changes to entropy. There are several challenges for the future, among them the exploration of how general is the arising of specific ‘control sites’ of allosteric cooperativity, the witness of the evolutionary record, the exploration of the effects of multiple mutations, and the contribution of the normal modes to allosteric systems that are also characterised by conformational change.
